# The Applications of Artificial Intelligence in Chest Imaging of COVID-19 Patients: A Literature Review

**DOI:** 10.3390/diagnostics11081317

**Published:** 2021-07-22

**Authors:** Maria Elena Laino, Angela Ammirabile, Alessandro Posa, Pierandrea Cancian, Sherif Shalaby, Victor Savevski, Emanuele Neri

**Affiliations:** 1 Artificial Intelligence Center, IRCCS Humanitas Research Hospital, via Manzoni 56, Rozzano, 20089 Milan, Italy; pierandrea.cancian@humanitas.it (P.C.); victor.savevski@humanitas.it (V.S.); 2 Department of Biomedical Sciences, Humanitas University, Via Rita Levi Montalcini 4, Pieve Emanuele, 20072 Milan, Italy; angela.ammirabile@humanitas.it; 3 Department of Radiology, IRCCS Humanitas Research Hospital, via Manzoni 56, Rozzano, 20089 Milan, Italy; 4 Department of Diagnostic Imaging, Oncological Radiotherapy and Hematology, Fondazione Policlinico Universitario Agostino Gemelli—IRCCS, 00168 Rome, Italy; alessandro.posa@gmail.com; 5 Department of Translational Research and New Technologies in Medicine and Surgery, University of Pisa, Via Roma 67, 56126 Pisa, Italy; master51087@gmail.com (S.S.); emanuele.neri@unipi.it (E.N.); 6 Italian Society of Medical and Interventional Radiology, SIRM Foundation, Via della Signora 2, 20122 Milano, Italy

**Keywords:** artificial intelligence, chest CT, chest X-ray, computed-aided diagnosis, COVID-19

## Abstract

Diagnostic imaging is regarded as fundamental in the clinical work-up of patients with a suspected or confirmed COVID-19 infection. Recent progress has been made in diagnostic imaging with the integration of artificial intelligence (AI) and machine learning (ML) algorisms leading to an increase in the accuracy of exam interpretation and to the extraction of prognostic information useful in the decision-making process. Considering the ever expanding imaging data generated amid this pandemic, COVID-19 has catalyzed the rapid expansion in the application of AI to combat disease. In this context, many recent studies have explored the role of AI in each of the presumed applications for COVID-19 infection chest imaging, suggesting that implementing AI applications for chest imaging can be a great asset for fast and precise disease screening, identification and characterization. However, various biases should be overcome in the development of further ML-based algorithms to give them sufficient robustness and reproducibility for their integration into clinical practice. As a result, in this literature review, we will focus on the application of AI in chest imaging, in particular, deep learning, radiomics and advanced imaging as quantitative CT.

## 1. Introduction

Severe Acute Respiratory Syndrome Coronavirus 2 (SARS-CoV-2) infection, named COVID-19 (coronavirus disease 2019), caused a global healthcare and economic crisis. The first cases were observed in Wuhan, China, in December 2019, and it rapidly spread across the world so that in early March 2020, the WHO decided to classify COVID-19 a pandemic.

Diagnostic imaging has a fundamental role in the clinical work-up of patients with suspected or confirmed COVID-19 infection, granting disease identification, screening and stratification based on the severity of lung involvement as well as in predicting the risk of complications and the need of intensive care unit (ICU) admission. Imaging helps, nonetheless, in the differential diagnosis of COVID-19 from other kinds of lung infections and diseases. However, due to the rapid diffusion of COVID-19 pandemic, a lot of hospitals and primary and secondary care structures found themselves unprepared, having trouble getting personal protective equipment (PPE) [1], thus making diagnostic imaging procedures difficult and risky to perform, [2] also considering the difficultly to fully and promptly clean the CT scanners between each examination.

In fact, imaging should be reserved to the following precise cases, as suggested in the advice guide for the diagnosis and management of COVID-19 by the WHO [3]:

- For the diagnostic workup of COVID-19 when RT-PCR testing is not available; when RT-PCR testing is available, but results are delayed; and when initial RT-PCR testing is negative, but with high clinical suspicion of COVID-19. In addition to clinical and laboratory data for patients with suspected or confirmed COVID-19, not currently hospitalized and with mild symptoms in order to decide on hospital admission/home discharge or on regular ward admission/intensive care unit admission.
- In addition to clinical and laboratory data for therapeutic management of patients with suspected or confirmed COVID-19, currently hospitalized and with moderate to severe symptoms.

Due to its high availability, portability and cost-effectiveness, chest X-ray (CXR) is the most widely used diagnostic imaging modality against COVID-19, contributing to the first assessment of patients with respiratory symptoms. Patients affected by COVID-19 can present with a pattern varying from normal lung to bilateral interstitial involvement, to opacification, based on the stage of the disease and the clinical presentation [4].

Chest computed tomography (CT) is usually performed in critically ill patients, in which there could also be the need to rule out pulmonary thromboembolism which can be a fatal complication of COVID-19 infection. CT imaging is more accurate than CXR, and is also used in cases of dubious finding at the radiographs: CT patterns are represented by peribronchial and peripheral ground-glass opacities (GGO), mostly basal and bilateral, with involvement of two or more lung lobes, with an increase in severity and consolidation and/or crazy paving pattern as the disease advances in the middle and late stages. However is important to outline that CT patterns of COVID-10 pneumonia are not specific, and superimposable to many other infectious and non-infectious pneumonia [5,6,7].

Lung ultrasound (US) does not have a clear role in the diagnostic approach to a suspected or confirmed COVID-19 case. Due to its great availability and mobility, it can be of great use for bedside evaluation of subpleural consolidations, pneumothorax and alveolar damage, even though its diagnostic accuracy greatly depends on the operator experience [8,9,10]. Recent progress has been made in diagnostic imaging with the integration of artificial intelligence (AI) with computer-aided design (CAD) softwares [11], leading to an increase in the accuracy of exams’ interpretation and to the extraction of prognostic information useful in the decision-making process [12,13,14,15].

Specifically, COVID-19 has catalyzed the rapid expansion in the application of AI to combat disease. As a result, previous authors made a summary of the work performed and the discriminatory ability of AI in its various diagnostic imaging applications.

Ghaderzadeh et al. in their systematic review analyzed papers published between 1 November 2019, and 20 July 2020 regarding the application of deep learning (DL) in chest X-ray and CT. In this review, they suggested that DL-based models share high accuracy in the detection and diagnosis of COVID-19 and that the application of DL reduces false-positive and negative errors compared to radiological examination performed by a radiologist [16].

Another review article by Shi et al. focused on the role of AI in chest CT and CXR in COVID-19 affected patients. They gave an overview of the whole pipeline regarding the implementation of DL in chest imaging, from image acquisition, segmentation to diagnosis, giving also insights regarding the follow-up and the public datasets available [17].

In this review, we explore the role of AI/ML in the diagnostic imaging of patients with COVID-19, including deep learning integration, radiomics features and quantitative CT imaging algorithms. We discuss its wide-range applications on the following domains:

Identification and screening of COVID-19 pneumonia,

For setting the differential diagnosis between COVID-19 pneumonia and other types of infectious pneumonia.

In the stratification and definition of severity and complications of COVID-19 pneumonia.

## 2. Search Strategy

Before setting up our search strategy we aimed at answering the following questions:

(1) What are the main indications for COVID-19 imaging?
(2) What is the workflow followed in image elaboration for AI solutions?
(3) Does DL improve the diagnostic abilities of radiologists in COVID-19 patients?
(4) What are the other applications of AI in COVID-19 patients (apart from the identification of the lesions?
(5) Are there any limitations for AI in this field?

After defining the aforementioned research question, we searched using the PubMed database by inserting the following keywords: “COVID-19,” “diagnosis,” “artificial intelligence,” “detection,” “chest x-ray,” “chest CT,” “deep learning,” “stratification,” “prognosis,” “differential diagnosis,” eventually, the related published studies were extracted and reviewed. We set inclusion criteria to refine the selection of manuscripts based on our subjective assessment of their relevance, novelty and being in English language.

## 3. Workflow of Images Segmentation, Annotation and Elaboration

Development of AI-based COVID-19 classification/segmentation models starts from their training with various images sources, usually represented by normal and abnormal (COVID-19, non-COVID-19) chest images. Data collection is, therefore, considered mandatory.

**Figure 1:**
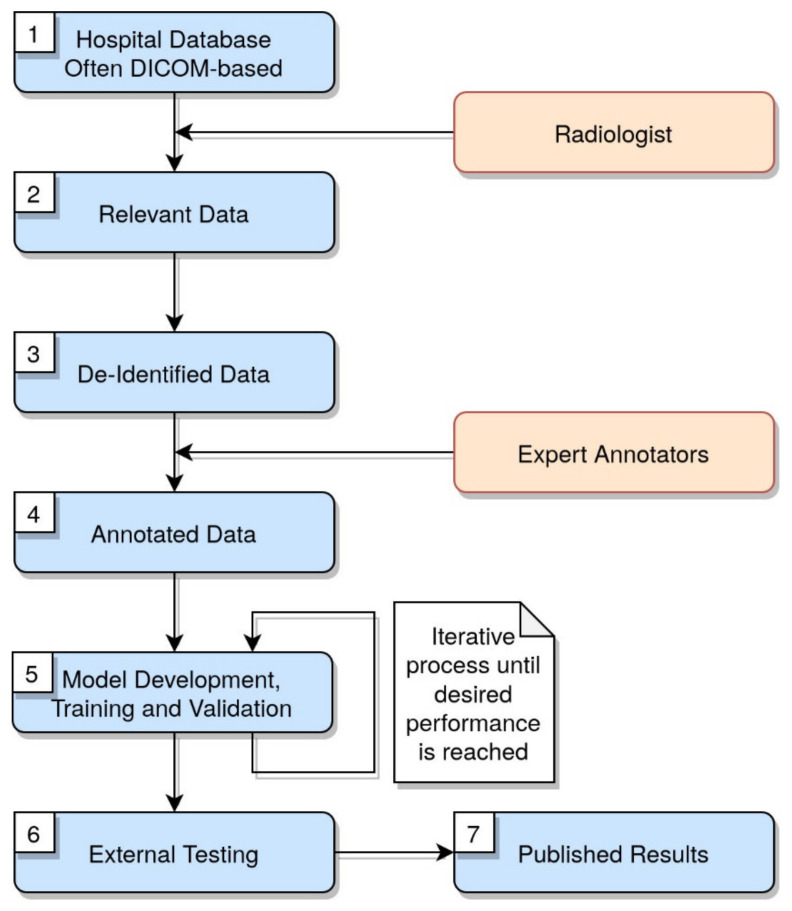
Workflow of image annotation, segmentation, and elaboration. The diagram illustrates the steps to follow when building a ML model using the radiological images.

The whole workflow of image annotation, segmentation, and elaboration is shown in Figure 1.

Patients’ data must be downloaded, queried, correctly de-identified and safely stored after ethical consent. The best approach to de-identification is pseudonymitazion; when the DICOM images are pseudonymized, the information that can point to the identity of a subject is replaced by “pseudonyms” or identifiers [18].

Manual selection of similar images according to basic criteria (age, technique, imaging findings) is always performed by expert radiologists to have the best training dataset. Image segmentation is a fundamental part of image processing and analysis for assessment of pathologic examinations. Segmentation is based on delineation of regions of interest (ROIs), as lung lobes, airways, focal or diffuse pathologies in the images [19,20,21,22,23]. A robust training model needs sufficient labeled images, which usually lack in case of COVID-19, mostly due to the time-consuming nature of this task in a pandemic setting; in these cases, the radiologist can be asked to interact with the segmentation network to supervise the machine learning methods [24]. An appropriate segmentation may help in monitoring the progression of COVID-19 pneumonia and the assessment of severity. AI models can be trained using available datasets or with the “transfer learning” method, making the most of already available models which also avoid mixing training and test data [25]. Features obtained from different convolutional neural network models can be classified with a support vector machine (SVM) classifier using images [26]. After training and testing, one or more other sets of images can be used for external validation of the model.

## 4. Artificial Intelligence in Chest X-ray

Several studies focused on the automatic classification of COVID-19 from CXR images [27,28,29,30,31,32,33,34,35], considering how useful it could be in emergency departments, urgent care, and resource-limited settings. Moreover, by matching CXR findings to clinical data prognostic models can be developed, to predict disease gravity, and stratify patients on the basis of their risk of developing severe disease and or complications.

### 4.1. AI in the Identification of COVID-19 Pneumonia at Chest X-ray

CXR can help in identify signs of pneumonia, also in case of negative RT-PCR test: sensitivity of CXR greatly depends on the stage of the lung infection and on the extent of the disease, as well as on the technical quality of the exam (usually performed bedside in critically ill patients), ranging from 50% to 84% [36,37,38]. Specificity is low, attested at 33% [36]. However, the COVID-19 pandemic kickstarted the development of AI-based models worldwide, for the automatic detection of pneumonia signgs on CXR images, which yielded great results: using automated machine learning algorithms and deep convolutional neural networks (DCNN), as well as deep transfer learning techniques, various Authors presented results in COVID-19 detection in which obtained a sensitivity ranging from 97.9% to 100%, a specificity between 95% and 98.8%, an accuracy ranging from 83.5% to 98%, and precision of up to 97.95% [27,35,39,40,41,42].

Accuracy can be improved by up to 99.41% when using support vector machines (SVM), which are supervised learning methods based on statistical learning theory [43] that work by dividing the dataset in training and test subsets [44,45], and up to 100% when using twice transfer learning (also known as transfer learning in three steps), and output neuron keeping (keeping output neurons that classify similar classes between the second and third step of the twice transfer learning), which improves training speed or performances particularly in the first phases of the training process [46]. Other approaches in COVID-19 pneumonia identification were performed using several convolutional layers and applying filters to each layer [33], as well as introducing stochastic pooling in DCNN [47], or using multiresolution approaches with improved results when compared to deep learning methods [48,49].

Moreover, Sahlol et al. used an efficient hybrid classification which adopted a combination of CNN and an improved swarm-based feature selection algorithm. This combination should achieve two main targets; high performance and resource consumption, storage capacity. In addition, they also proposed a novel robust optimizer called Fractional-order Marine Predators Algorithm (FO-MPA) to efficiently select the huge feature vector produced from the CNN. Then, they tested and evaluated the proposed approach by performing extensive comparisons to several state-of-art feature selection algorithms, most recent CNN architectures and most recent relevant works and existing classification methods of COVID-19 images [50].

**Figure 2:**
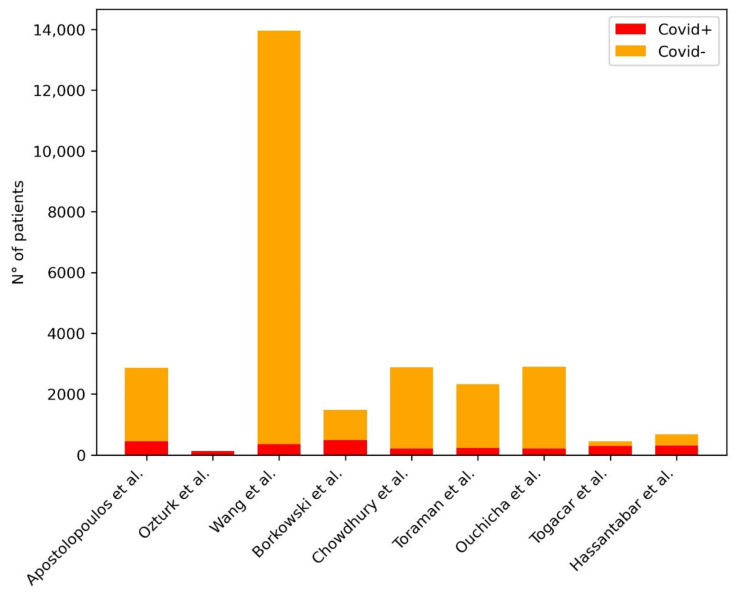
Distribution of subjects included in the studies for the development of ML models for the diagnosis of COVID-19 pneumonia at CXR. The plot shows the distribution of the subjects included in the studies: in the legend in the right upper corner of the figure, the red bar represents the COVID-19 pneumonia group of patients, the yellow bar represents the non-COVID-19 pneumonia group of patients.

Table 1 provides a summary of the papers included in the review, focused on AI in the identification of COVID-19 pneumonia signs at CXR. Figure 2 shows the distribution of subjects included considering those studies where it was clearly stated.

### 4.2. AI in the First Assessment of COVID-19 Pneumonia at Chest X-ray

As CXR is often the first-line diagnostic imaging modality when facing a patient suspected of COVID-19 infection, even if less sensitive than lung CT, it plays a great role in the first assessment of patient. Even though the confirmation of COVID-19 infection should always come from RT-PCR tests performed on naso-pharyngeal swabs [51], these tests could not be readily available and may take time to give the result; therefore, a rapid CXR assessment of patients with respiratory symptoms should be performed, and AI can play an important role, especially when dealing with a large number of requests in the emergency settings [52]. Most literature studies use AI in CXR to distinguish between COVID-19 and other pneumonia and healthy patients [53,54,55]. Xia et al. described the use of a rapid and economic classifier for screening of COVID-19 from influenza-A/B pneumonia which combined CXR (or CT-localizer scanogram) data with clinical features, with 91.5% sensitivity and 81.2% specificity and an AUC of 0.971 (95% CI 0.964–0.980) [56].

**Figure 3:**
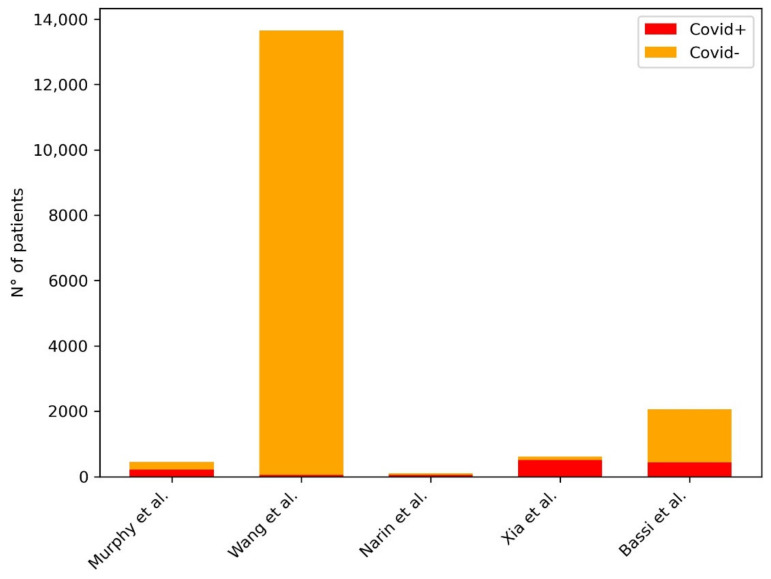
Distribution of subjects included in the studies for the development of ML models for the screening of COVID-19 pneumonia at CXR. The plot shows the distribution of the subjects included in the studies: in the legend in the right upper corner of the figure, the red bar represents the COVID-19 pneumonia group of patients, the yellow bar represents the non-COVID-19 pneumonia group of patients.

In Table 2, we provided a summary of the papers included in our review focused on AI in the screening of COVID-19 pneumonia at Chest X-ray. Figure 3 shows the distribution of subjects included considering those studies where it was clearly stated.

### 4.3. AI in the Stratification and Definition of Severity and Complications of COVID-19 Pneumonia at Chest X-ray

As diagnostic images in COVID-19 correlate with disease severity, AI can be used as a prognostic tool, helping monitoring disease evolution and course, and identifying patients at risk of ICU admission [57,58]. However, there is no standardized method in reporting CXR findings in terms of disease severity. Li et al. used the pulmonary x-ray severity (PXS) score, a DL-based algorithm providing quantitative measures of COVID-19 severity on CXR, as an adjuvant tool to radiologists’ work—which, however, always decided on the severity grading and definitive radiological report-, and noticed an improvement in the assessment of the severity on a 4-point scale (normal/minimal, mild, moderate, severe) and in the inter-reader agreement, with no need for radiologists’ training on the use of the score [59,60]. Li et al. also found that the severity scores were significantly associated with intubation/death within 3 days from the admission, in CXR rated moderate or severe [59]. Mushtaq et al. reported in their retrospective study that an AI-powered severity score based on the percentage of pixels involved by opacity or consolidation for each lung at the CXR, adjusted at the multivariate analysis for demographics and comorbidities, showed that a value ≥30 at the hospital admission CXR was an independent predictor for mortality and ICU admission for COVID19 (*p* < 0.001), and found a significant link with admission pO2/FiO2 levels [61]. Zhu et al. compared the evaluation of an AI algorithm to the one performed by independent expert radiologists on the results of CXR in patients suspected for COVID19 in terms of disease severity using criteria based on the degree of lung opacity and geographical extent of the opacity, finding a strong correlation between the two severity scores [62].

**Figure 4:**
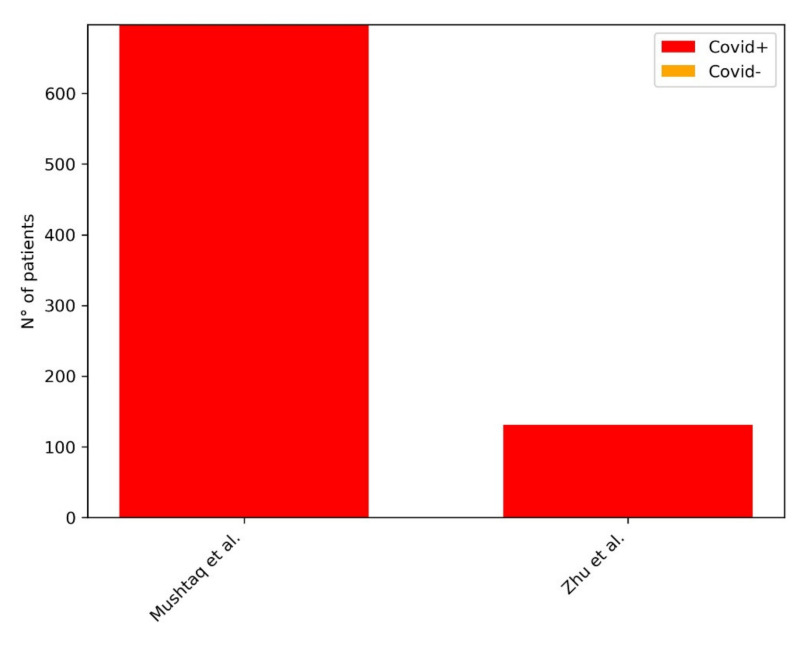
Distribution of subjects included in the studies for the development of ML models for the stratification and definition of severity and complications of COVID-19 pneumonia at CXR. The plot shows the distribution of the subjects included in the studies: in the legend in the right upper corner of the figure, the red bar represents the COVID-19 pneumonia group of patients.

Table 3 provides a summary of the papers included in our review focused on AI in the stratification and definition of severity and complications of COVID-19 pneumonia at CXR. Figure 4 shows the distribution of subjects included considering those studies where it was clearly stated.

### 4.4. AI in the Differential Diagnosis of COVID-19 Pneumonia from Other Pneumonia at Chest X-ray

Various authors also investigated the effectiveness of supervised AI learning models in aiding medical professionals in the differential diagnosis between COVID-19 pneumonia and other lung diseases, in particular the non-COVID-19 viral pneumonia, with a reported accuracy of up to 87% [33,39,41,42,63,64]. Jin et al. proposed a three-step hybrid model, incorporating a feature extractor, feature selector, and an SVM classifier, reporting an overall accuracy rate of 98.6%, with a remarkable reduction of training time and of the training sets size [65].

However, the differential diagnosis is impaired by the aspecific picture of COVID-19 pneumonia, similar to other viral and non-viral interstitial diseases. AI models should be adequately trained to achieve state-of-the-art diagnostic efficacy in the external validation process and in the real-life radiological workflow: CXR obtained in different views (postero-anterior (PA), latero-lateral, as well as bedside ones) must be differentiated, and the same goes for age groups, distinguishing pediatric patients from adults. Some authors chose to train models only on PA views, as it is usually the most common view used in the emergency department, even though bedside CXR are getting more and more important in the first diagnosis and in monitoring critically ill patients [66,67].

AI evolution could aim to help the diagnostic radiology in screening, diagnosing and grading CXRs, even though there are serious concerns on the potential risk of this situation happening [68].

Table 4 provides a summary of the papers included in the review focused on AI in the differential diagnosis of COVID-19 pneumonia from other pneumonia at Chest X-ray.

## 5. Artificial Intelligence in Chest CT

Machine learning approaches applied to CT images in COVID-19 pneumonia show great potential for improving diagnostic accuracy as well as for the prediction of patient outcomes and many studies have been focused on this topic.

Indeed, AI takes advantage of the large quantity of imaging data that can be used to train algorithms, and if effective, it could bring to a revolution in the identification and triage of patients with suspected COVID-19.

### 5.1. AI in the Identification of COVID-19 Pneumonia and Its Complications at Chest CT

From the beginning of the COVID-19 pandemic, the use of AI for detection of the radiological signs of pneumonia on CT imaging has been investigated, also in cases of false-negative results at RT-PCR [69], and augmented radiologists workload [70].

Considering the central role of imaging in the management of infected patients, multiple deep-learning algorithms have been developed to face the increased needs, also within just 10 days [71]. A pilot study by Yang et al., performed in the first two months of 2020, evaluated the performance of a DenseNet algorithm model—an improved CCN—for COVID-19 detection on HRCT. It yielded an AUC of 0.98 and a sensitivity of 97%, but an accuracy of 92% and specificity of 87% resulted slightly lower than those of an experienced radiologist. The authors concluded that their DL model had a human-level performance and allowed to save time due to a rapid diagnosis in about 30 s versus 5–10 min needed by a radiologist. A limitation of this study was a restricted number of included patients (146 with COVID-19 and 149 controls), further divided into training, validation and test sets [72].

To overcome this limit, multiple studies utilized datasets composed of thousands of patients derived from public sources or as occurred in multicenter trials. Therefore, Harmon et al. analyzed a heterogeneous multinational CT dataset composed of 2617 patients, overcoming a limited applicability to different populations, demographics or geographies, and maximizing the potential for generalizability. The 922 included cases of COVID-19 were from China, Italy and Japan, while the balanced control population was identified either from 2 US institutions or from a publicly available dataset (LIDC). Their image classification model used both hybrid 3D and full 3D models based on a Densnet-121 architecture, and they achieved a 0.949 AUC, resulting in 90.8% accuracy for COVID-19 identification on chest CT [73].

In addition to public datasets, previously validated AI algorithms are available for further confirmation of their performance or as assistant tools to clinicians and radiologists [74]. In this regard, Chen et al. created a cloud-based open access AI platform to improve the diagnosis of COVID-19 pneumonia. They developed a UNet++-based model with an accuracy of 96% for COVID-19 detection on HRCT in multiple testing datasets, either internal (retrospective and prospective) and external ones. Furthermore, the use of a similar deep-learning based model has the potential to reduce the number of missed diagnosis, especially in early phases, because the lung infection foci could be mild and need observation under 0.625-mm layer scanning [75].

Other authors focused not only on the pneumonia detection on a CT scan, but also on a quantitative assessment [74]. In fact, Zhang et al. analyzed images from 2460 patients using the uAI Intelligent Assistant Analysis System (a modified 3D CNN and a combined V-Net with bottle-neck structures) to segment anatomical lung structures and to accurately localize infected regions, according to the specific lobes and segments. Their findings were consistent with those of previous studies [76] that demonstrate a typical bilateral involvement, mainly in the dorsal segments, with GGOs as the most common CT feature [77].

These results have been confirmed also in other studies about the role of quantitative CT [78]. Du et al. evaluated pre-discharge CT scans in asymptomatic patients with negative RT-CR with an AI-assisted system (InferRead CT pneumonia software). Their quantitative image analysis resulted in a prevalence of fibrosis as the second common manifestation after GGOs, characterized by heterogeneous density and rigid reticulation [79].

To ease the evaluation of COVID-19 patients according to the findings on chest CT scan, the standardized score CO-RADS has been introduced to grade the level of suspicion from very low (1) up to very high (5), providing a higher performance in patients with moderate and severe symptoms (average AUC 0.91 for predicting RT-PCR outcome and 0.95 for clinical diagnosis) and a higher interobserver agreement for categories 1 and 5 [80]. Lessmann et al. aimed to develop a CO-RADS AI system to obtain an automated assessment of the suspicion value. CO-RADS AI included three deep-learning algorithms based on a U-Net architecture that automatically performed lobe and lesion segmentation, prediction of a CT severity score according to the percentage of affected parenchymal tissue per lobe and, at last, the assignment of the CO-RADS value. The key result of this study was a high diagnostic performance in the identification of COVID-19 patients with an AUC curve of 0.95 in the internal test set and of 0.88 in the external cohort [81]. However, its use is controversial because it does not take into consideration clinical and laboratory findings to build a diagnosis of COVID-19, also AI-assisted.

In fact, a study by Liu et al. demonstrated that a combined clinical-radiological model outperformed the CO-RADS and a clinical model in the COVID-19 diagnosis. Their preliminary study investigated the performance of a combined radiomics model that included 5 clinical features and a radiomic signature, after multivariate logistic regression analysis: age, lesion distribution (central or peripheral), neutrophil ratio, lymphocyte count, CT score and mean Radscore. The latter was calculated by 8 radiomic features, selected after the application of a mRMR algorithm and LASSO logistic regression algorithm. The result was an open-source constructed radiomics model with an AUC of 0.98, sensitivity of 0.94 and specificity of 0.93 [82]. Similar results have been achieved in another study that confirmed a mixed model—presented as nomogram—as the highest predictor of COVID-19 with an AUC of 0.955 (versus an AUC of 0.626 of the clinical model). It included either CT characteristics of the lesions (distribution, maximum lesion range, involvement of lymph nodes and pleural effusions) and a RadScore based on a signature of 3 features selected by LASSO regression [83].

Another use of radiomic models has been described in the non-invasive monitoring of ARDS, a life-threatening COVID-19 complication. Indeed, Chen et al. compared the performance of traditional quantitative and radiomics analysis of CT images. While the former quantified the infected regions through the calculation of volume and percentage of infection, the latter included 30 radiomic features selected by regression analysis and combined into a risk score. Results showed that the radiomics model was the most promising one because of the highest accuracy and specificity, despite a similar AUC of 0.94. According to the authors, sensitivity is more important than specificity in an ARDS screening due to the high risk related to delayed oxygen treatment in false-positivity results [84].

Voulodimos et al. adopted a semantic segmentation approach, which can be implemented in a two-step process: (i) feature extraction over an image patch and (ii) a training process, using annotated datasets. Using this method, each pixel is described by feature values, extracted locally, over a, typically, small area, denoted as “patch”. Deep learning approaches do both steps for a given set of data [85].

The possibility of segmentation transferability in COVID-19 CT has been investigated by Wang et al. They presented a set of experiments to better understand how different non-COVID19 lung lesions influence the performance of COVID-19 infection segmentation and their different transfer ability under different transfer learning strategies. They concluded clear benefits of pre-training on non-COVID19 lung lesion datasets when public labeled COVID-19 datasets are inadequate to train a robust deep learning model [86].

Saood et al. proposed a new fully automated deep learning framework for rapid quantification and differentiation between lung lesions in COVID-19 pneumonia on both contrast and non-contrast CT images using convolutional Long Short-Term Memory (ConvLSTM) networks. They showed a strong agreement between expert manual and automatic segmentation for lung lesions; describing excellent correlations of 0.978 and 0.981 for ground-glass opacity and high opacity volumes [87].

Akram et al. presented a novel entropy-based fitness optimizer function implementation, which selects the chromosomes with maximum information. The only chromosome with maximum fitness value is selected to get the sub-optimal solution in the minimum number of iterations. To conserve maximum information and to obliterate the redundant features at the initial level, a preliminary selection process is initiated on each feature set using the entropy-controlled fitness optimizer. To exploit the complementary strength of all features, a feature fusion approach is utilized which combines all the competing features to generate a resultant feature vector. The previously adopted methods of machine learning utilize either sole or hybrid approaches for feature extraction. Though both methods have their advantages and drawbacks, but the fused feature space has more capacity to retain the dexterous features. Due to this flexibility, the hybrid approaches have gained much popularity among the researchers. However, selection of the most appropriate feature extraction technique is quite a sensitive task, which needs to be handled carefully, otherwise, it may result in feature redundancy and, therefore, increased correlation. In this work, they utilized four different techniques—belongs to two different categories, statistical and texture. Two feature families were not considered, color and shape, because of their limited impact and significance in this application. Using the proposed framework, the achieved accuracy using the Naive Bayes classifier is 92.6%, 92.6%, whereas other classifiers (EBT, L-SVM and F-KNN) behave significantly better to achieve an average accuracy of 92.2%, 92.1%, 92.2%, 92.1% and 92.0%, 92.0%, respectively. From the sensitivity and specificity values, the proposed framework was successfully managed to achieve high true positive and negative rates [88].

Mukherjee et al. developed a CNN-tailored DNN for COVID-19 diagnosis, integrating either CT and CXR images. Their proposed DNN based on a mixed database of integrated modalities reached an AUC of 0.9808, higher than those of other existing DNN (Inception, MobileNet and ResNet). Moreover, the performances score using separate dataset appeared to be higher for CXRs with an AUC of 0.9908 vs. 0.9731 for CT scan [89].

**Figure 5:**
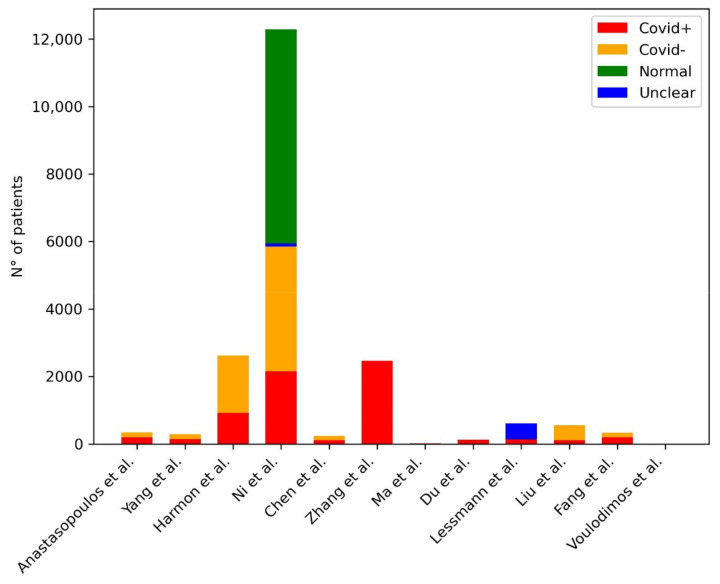
Distribution of subjects included in the studies for the development of ML models for the diagnosis of COVID-19 pneumonia at Chest CT. The plot shows the distribution of the subjects included in the studies: the red bar represents the COVID-19 pneumonia group of patients, the yellow bar represents the non-COVID-19 pneumonia group of patients, the green bar represents the group of healthy patients, the blue bar represents the group of patients for which their health status was unclear.

Table 5 provides a summary of the papers included in the review focused on AI in the diagnosis of COVID-19 pneumonia at Chest CT. Figure 5 shows the distribution of subjects included considering those studies where it was clearly stated.

### 5.2. AI in the Screening of COVID-19 Pneumonia at Chest CT

The application of AI to CT images for the immediate triage of COVID-19 patients may be of assistance due to delayed results of RT-PCR as definitive viral testing.

Javor et al. used an open-source data of 6868 CT images to train their CCN model ResNet50 that achieved high accuracy with an AUC of 0.956, higher than those of radiologists. They described the importance of the ML model in the patient triage for the possibility to identify rule-in and rule-out thresholds for COVID-19 diagnosis, compared to a dichotomous decision of radiologists. In case of high level of suspicion, the patient should be isolated until the confirmation of rejection by an RT-PCR test [90]. However, CT scan may have a low negative predictive value, especially in early phases of the disease. A joint AI algorithm that integrated chest CT findings and clinical history enabled a rapid diagnosis of COVID-19 with an AUC of 0.98 that might have a fundamental role in the triaging, allowing rapid isolation of infected people and avoiding delayed treatments. The evaluated model was first developed on a CNN to learn imaging characteristics on initial CT scans and then on a MLP to classify patients according to the clinical information (sex, age, exposure history, clinical symptoms—fever and cough—and laboratory findings—WBCs). Finally, a neural network model combined radiological and clinical data to predict COVID-19 status [91].

Another study performed in an emergency department confirmed the positive performance of a mixed predictive ML model in the triage. It was based on the CO-RADS score from chest CT and additional data—laboratory findings (ferritin, leukocytes, CK), diarrhea and number of days from onset of the disease. The added value of the prediction model compared with CT alone was increased AUC (0.953 vs. 0.930) and accuracy (93.1% vs. 90.4%), probably due to specific laboratory anomalies. Nevertheless, authors concluded that 9% of the included patients with positive RT-PCR were false negative according to the prediction model and the nasopharyngeal swab should be the primary standard test [92].

**Figure 6:**
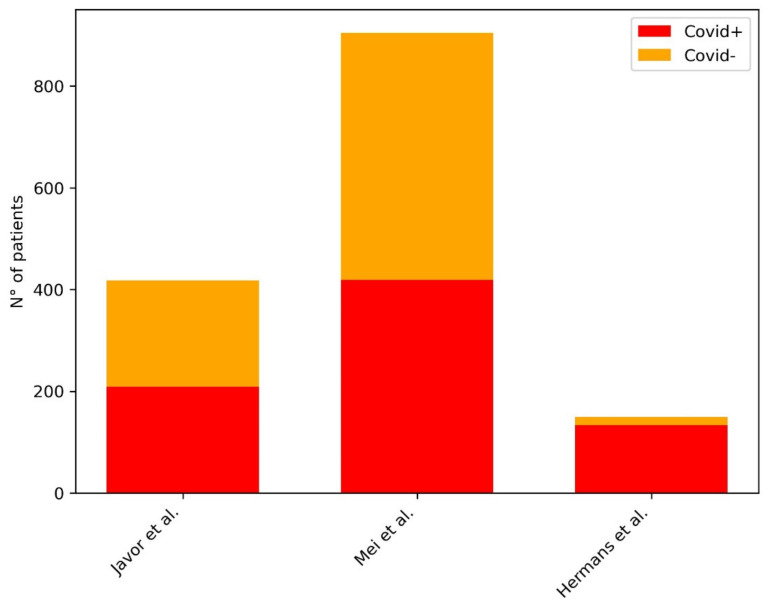
Distribution of subjects included in the studies for the development of ML models for the screening of COVID-19 pneumonia at Chest CT. The plot shows the distribution of the subjects included in the studies: in the legend in the right upper corner of the figure, the red bar represents the COVID-19 pneumonia group of patients, the yellow bar represents the non-COVID-19 pneumonia group of patients.

In Table 6, we provided a summary of the papers included in our review focused on AI in the screening of COVID-19 pneumonia at Chest CT. Figure 6 shows the distribution of subjects included considering those studies where it was clearly stated.

### 5.3. AI in the Stratification and Definition of Severity and Complications of COVID-19 Pneumonia at Chest CT

Different studies have already demonstrated the correlation between conventional CT scores and prognosis of COVID-19 patients, using semi-quantitative methods based on visual scores [93,94,95]. As an attempt to avoid subjective and time-consuming evaluations, multiple AI models have been developed and tested to accurately stratify patients into severity stages and to improve the clinical decision-making process. According to the ATS, the major criteria for the definition of severe pneumonia are respiratory failure in need for mechanical ventilation (MV) or septic shock treated with vasopressors; other minor criteria include increased respiratory rate (>30/min), P/F ratio < 250 or hypotension requiring fluid resuscitation [96]. Therefore, these are the most common endpoints used to find potential high-risk patients.

According to Chatzitofis et al., a VoI aware DNN could assess patients’ conditions and prognosis even without results of laboratory tests, as occurred shortly after the ED admission. They introduced a two-stage data-driven approach to classify patients into three classes—moderate, severe and extreme, considering their risk to be discharged, hospitalized or admitted to ICU, respectively. The proposed algorithm was trained with a COVID-19_CHDSET Dataset, composed by CT images from Milan, an extensively involved area during the first months of the COVID-19 pandemic. The DenseNet201-VoI model reaches an AUC of 0.97, 0.92 and 1.00 for the three groups, respectively, and accuracy of 88.88%, specificity of 94.73% and sensitivity of 89.77% [93]. Xiao et al. developed and tested a DL-based model using multiple instance learning and CNN (ResNet34) on CT imaging. It resulted in an excellent performance for the prediction of disease severity (AUC of 0.892) that is, in turn, positively correlated with area and density of lung lesions. Moreover, the clinical significance of the model relied on the possibility to identify mild disease in early stages that could progress to a more severe form, characterized by a lower survival probability [97].

The idea of a possible rapid deterioration of mild cases has been further analyzed by Zhu et al. whose joint regression and classification model was able to predict the conversion time from a mild to a severe case in a unified framework with a sensitivity of 76.97% and an average conversion time of 4.59 days [98]. Another fully automated DL-model succeeded in diagnostic and prognostic analysis of COVID-19, after training in a large dataset of 4106 patients. Authors defined the length of hospital stay as prognostic end event, knowing that longer hospitalization might imply worse prognosis and longer recovery time. COVID-19Net showed a good diagnostic and prognostic performance in the stratification of low- and high-risk patients with significant differences in days of hospital stay [99].

A DL prognostic model developed by Meng et al. predicted the probability of patients’ death within two weeks. This 3D-CNN De-COVID19-Net outperformed clinical, radiomics-based and pure CNN models (without incorporation of the clinical model) with an AUC of 0.943 in the identification of high-risk patients, i.e., died within 14 days, that required more intensive care [100].

Specific laboratory measurements can be combined with CT features to create AI-based prediction models for the stratification of severe patients, as demonstrated by Li et al. (AUROC of 0.93). They segmented CT imaging through a deep CNN to extract essential features and selected 12 laboratory tests that showed the largest change in the two groups of patients, mainly D-dimer, LDH and lymphocytes as predictors of higher mortality risk. Moreover, lymphocytes, neutrophils, D-dimer and platelets-large cell ratio demonstrated a significant correlation with selected CT features [101].

An additional DL model mixed an artificial neural network (ANN) for clinical and laboratory data and a CNN for 3D CT imaging data to classify patients in high risk of severe progression (event) or low risk (event-free). The considered events included respiratory deterioration (high-flow nasal cannula, MV, ICU admission), septic shock, renal failure or death. In the correlation heatmap of clinical and laboratory features, CRP and WBC had a strong positive correlation with the endpoint, age was described as significant risk factor related to the endpoint; oxygen saturation and female sex were negatively correlated with the endpoint. This mixed ACNN model obtained a high performance with an AUC of 0.916, accuracy of 93.9% and specificity of 96.9% [102].

An approach to estimate the prognostic utility of CT findings is based on a quantitative image assessment, using computer-aided software for segmentation and quantification of lung volumes according to different Hounsfield Unit (HU). Hu et al. performed a pilot study in the first two months of 2020 to demonstrate the validity of quantitative CT images in the evaluation of CT findings between mild and severe patients. They discovered a prevalence of consolidative and progressive lesions (crazy paving and “white lung”), mainly in lower lobes, in the severe group of patients, using a total lung and a per-lobe severity score to estimate pulmonary involvement and a 2D UNet model for the automatic lesion segmentation. However, this cross-sectional study lacked analysis of follow-up images, considering that the analysis of dynamic CT images could be useful for prognostic purposes [103].

Therefore, a Chinese retrospective study quantitatively evaluated lung involvement on serial CT scan with a deep-learning model, tracking the modification of the percentage of lung opacification (QTC-PLO) as a unique parameter. Authors divided the 126 included patients into four categories (mild, moderate, severe and critical) according to clinical features at baseline; they underwent at least two CT scans as inclusion criteria (median interval between baseline and first follow-up: 4 days) and, eventually, a second follow-up CT. The study results showed a significant difference in QTC-PLO among clinical groups at baseline (0%, 2.2%, 28.9%, 49.6%, respectively) with a sustained progression of imaging findings at first follow-up CT (median: 3.6% vs. 8.7%) and a plateau on second follow-up CT [19].

Similarly, Li et al. developed a fully automated AI system using a U-Net structure to assess disease severity and progression in severe and non-severe patients, considering the portion of infection (POI) and the average infection HU (iHU) in longitudinal CT scans. The two imaging biomarkers reached an AUC of 0.97 for POI and 0.69 for iHU and significant difference in the two severity states; authors concluded that only POI can be considered an effective indicator of COVID-19 severity taking into consideration high specificity and sensitivity; iHU could be affected by respiratory status and reconstruction slice thickness [104].

Zhang et al. analyzed temporal changes of quantitative lung lesion on CT scan from the onset of symptoms in common and severe groups, according to percentages of GGO-volume (PGV), consolidations (PCV) and total lesions (PTV). The used AI system combined the CNN and thresholding methods for lung segmentation and detection of patchy shadows, followed by automatic calculation of quantitative features by AI algorithms. Severe patients exhibited greater PGV, PCV and PTV in all the 5 stages of the diseases (0–30 days), a longer time to peak (17 vs. 12 days, respectively) and a higher peak percentage (22–25% vs. 2.5–5%, respectively) and longer recovery time [105].

Similar results have been demonstrated by Pan et al. that predicted a faster peak in moderate group compared to severe group (18 vs. 23 days, respectively, from onset of symptoms) with faster lesions absorption. Moreover, their DL model COVID-Lesion Net showed a good correlation with conventional CT scores (Spearman’s correlation coefficient 0.920) [106].

Other authors focused on the correlation between quantitative CT data with clinical features or laboratory values. Cheng et al. employed a uAI Discover-2019nCoV software to quantify images and to report a positive correlation between quantitative parameters (GGOs, consolidations and total lesions) and CRP, ESR and a negative correlation with lymphocyte count. Then, the proportion of total lesions resulted positively correlated with LDH [107].

An Italian retrospective study proved similar correlations, extending their results to parameters related to respiratory function (PaO_2_, pH, HCO^3−^, P/F). In fact, all the 108 included were in need for supplemental oxygen with NIV, CPAP or IV by ET. Their semi-automatic software showed a strong negative correlation between P/F ratio or hypercapnia, expression of hypoxia, and analyzed CT volumes. [108] Moreover, the Dense-UNet used by Mergen et al. further confirmed the previously described positive correlations about CRP and leukocytes. Authors underlined the negative correlation between percentage of opacity (PO) or percentage of high opacity (PHO, consolidations) with SO_2_ as an additional demonstration that patients in need for supplemental oxygen have a higher proportion of involved lungs [109].

In this regard, multiple studies have examined the utility of radiographic findings for the prediction of respiratory deterioration and consequent ICU admission by a quantitative CT analysis. A single-center retrospective study by Lanza et al. explored the role of quantitative computer-aided CT analysis as outcome predictor. The compromised lung volume (%CL), sum of poorly aerated and non-aerated parenchyma (from −500 to 100 HU), could predict oxygenation support, either low- and high-flow (%CL 6–23%, AUROC 0.83), and intubation (%CL > 23%, AUROC 0.86); moreover, %CL shown a negative correlation with P/F ratio, sign of deterioration of respiratory function, and was predictive of in-hospital mortality (HR 1.02) [110].

Similar results have been obtained in a retrospective study that confirmed the AI-calculated percentage of total opacity >51% as the main predictor for MV (AUC 0.87) and all-cause mortality during hospitalization (AUC 0.88). Moreover, they proposed a prognostic model that included biochemical variables (LDH level for mortality and troponin I for MV) and imaging data (total opacity for mortality and CT severity score for MV) with a good risk classification of hospitalized patients. [111] A multiparametric model of imaging-derived features—affected lung volume—and inflammatory laboratory parameters—CRP and IL-6—has been tested in a German Cohort to estimate the need for ICU treatment. The multivariate random forest modelling showed an AUC of 0.79, sensitivity of 0.72, specificity of 0.86 and accuracy of 0.80; affection of upper lung lobes could be considered an important parameter in the risk estimation (mean importance 0.184) [112].

Liu et al. proved that the quantitative CT evaluation with radiographic changes in the firsts 4 days after admission had excellent predictive capability (AUC 0.93) for severe disease, outperforming APACHE-II, NLR and D-dimer. The AI algorithms calculated percentages of GGOs (PGV), consolidation (PCV) and semi-consolidation (PSV) [113]. A further retrospective study assessed the feasibility of an automated quantification process of GGOs (−700–−501 HU), one of the most significant lesions of COVID-19 pneumonia, and normally restricted parenchyma (−900—−701 HU). They affirmed that GGOs could be an objective biomarker for lung injury due to a statistically significant correlation between the measured volumes and a respiratory assessment severity score on 6 categories, from absence of hospitalization and inability to resume normal activity (1) to death (7) [114]. On the other hand, a software-based quantitative CT assessment of the normal lung parenchyma percentage (SQNLP) has proven to accurately predict ICU admission if <81.1% (sensitivity 86.5% and specificity 86.7%). Furthermore, SQNLP <82.45% can show severe pneumonia with a sensitivity 83.1% and specificity 84.2%, characterized by increased presence of crazy-paving pattern (specificity 97.2%) [115]. Wang et al. focused on the risk of ARDS, primary cause of ventilation in COVID-19 patients. Their retrospective study used a Vb-Net model to segment lesions, discovering that the proportion of specific lesion density in the range −549–−450 HU was at high-risk for ARDS. In fact, total volume and average density of lung lesions were not statistically related to ARDS [116].

Radiomics analysis represents an additional approach to predict prognostic outcome of COVID-19 patients. A first attempt has been made to quantitatively analyze pulmonary lesions, dividing them in mild (Grade I) or moderate/severe (Grade II). After features preselection with a LASSO algorithm, the radiomic signature was built with 9 features and it achieved an AUC of 0.87 in the test set. The impact of the grading regards the subsequent treatment strategies, because mild lesions usually need only supportive treatment, while more severe ones need symptomatic treatment, up to invasive ventilation [117].

In a similar way, a tested radiomic model can predict not only the extent of pulmonary opacities (AUC 0.99), but also the type of lesions (0.77). In this case, skewness and small-area low gray-level emphasis were the best indicators of GGOs, considering that the category of pulmonary opacities has an important role in the pneumonia severity in addition to the volume of affected parenchyma [118].

Fu et al. performed a retrospective study in a cohort of patients divided into stable and progressive groups according to clinical manifestations, laboratory tests and CT imaging findings (statistically significant number of lesions). They tested the discriminatory capacity of a radiomic signature of 7 features, after application of mRMR and LASSO algorithms, with significant differences in the RadScore of the 2 groups. Moreover, cough and abnormal CRP values could improve the detection of patients in the progressive group [119].

In fact, other studies have reported an improved performance of their radiomic nomogram in the prognosis prediction after the integration of clinical factors. An example is those described in the retrospective analysis by Chen et al., composed of a Radscore of 15 features integrated with clinical information (age, gender, neutrophils count, % of NK cells and CD3) [120].

Wu et al. demonstrated that the integration of a radiomic signature with clinical risk factors (age, sex, type on admission, comorbidities) is more important in the early phases of COVID-19 for its accurate prediction of poor outcome (death, MV, ICU admission) with an AUC of 0.862 (vs. AUC of 0.816 of the RadScore alone) [121].

A peculiar merged model based on 6 significant radiomic features and DL model based on 3D-Resnet-10 has been analyzed to distinguish severe and critical cases of COVID-19. In the test cohort, the merged model yielded an AUC of 0.861, compared to AUC of 0.838 and 0.787 of single radiomic and DL models respectively, demonstrating the complementarity between the two types of features [122].

A Chinese retrospective multicenter study showed accuracy in the prediction of hospital stay in COVID-19 patients, as predictor of patients’ prognosis. Authors determined 10 days as the optimal cut-off value, classifying patients into short-term (<10 days) and long-term (>10 days) hospital stay. Their radiomic models of 6 features were based on logistic regression (LR) and random forest (RF) and reached an AUC of 0.97 and 0.92, respectively [21].

Differently from the previous studies about the analysis of the focus of pneumonia for patient stratification, Tan et al. tested their radiomics automatic ML model on the non-focus lung areas in the first CT scan of COVID-19 patients because they affirmed it could be difficult to distinguish initial areas of interstitial inflammation by eyes in early CT images. Authors included 219 first chest CT of patients with moderate and severe symptoms from which they extracted image texture features to construct classification models. The proposed model demonstrated a good prediction of COVID-19 pneumonia and its different clinical types due to differences in the non-focus areas with an AUC > 0.95 [21].

Moreover, a radiomic model combining CT feature and clinical data has been tested for its role in the prediction of RT-PCR negativity in order to identify the right retesting time. In this way, it is possible to avoid unnecessary repeated tests and prolonged hospital stay. Cai et al. included 203 patients in their retrospective study, divided into RT-PCR negative and RT-PCR positive groups according to the results of 3 RT-PCR tests performed after 3–5 days from symptoms disappearance. For each patient, 20 different features (clinical, quantitative and radiomic) were collected and compared between the two groups. Authors concluded that the RT-PCR negative group had a longer interval from onset of symptoms to CT scan (23 vs. 16 days) and the radiomic model of 9 features had a good performance for differentiating the RT-PCR negative group with an AUC of 0.812 [123].

Among the risk factors for severe COVID-19, comorbidities have been associated with increased risk of progression, probably due to a persistent pro-inflammatory state and attenuation of the immune response [124].

Lu et al. analyzed the effect of diabetes mellitus on chest CT features and COVID-19 severity in 3 groups of patients divided according to their clinical history of DM and HbA1c level. Their CT images were quantitatively evaluated, focusing on percentage of total lung lesion volume (PLV), percentage of ground-glass opacity volume (PGV) and percentage of consolidation volume (PCV) as parameters of pneumonia severity.

It was demonstrated a positive correlation between blood glucose level, measured also with blood fasting glucose, at admission and pulmonary involvement (higher PLV, PGV and PCV) that, in turn, were predictors of poor clinical outcomes (AUC of 0.796, 0.783, 0.816, respectively) [125]. Another retrospective study quantified pneumonia lesions on CT images through a UNet neural network to assess the influence of comorbidity on COVID-19 patients.

Differently from the previous study, Zhang et al. included hypertension—the most common, COPD and cerebrovascular diseases in addition to DM, already described as major risk factors [126]: authors found a significant correlation with age, length of incubation period, abnormal laboratory findings and severity status. Moreover, a higher number of comorbidities resulted in a higher number of CT lesions, especially in presence of DM as main risk factors for lung volume involvement [127].

**Figure 7:**
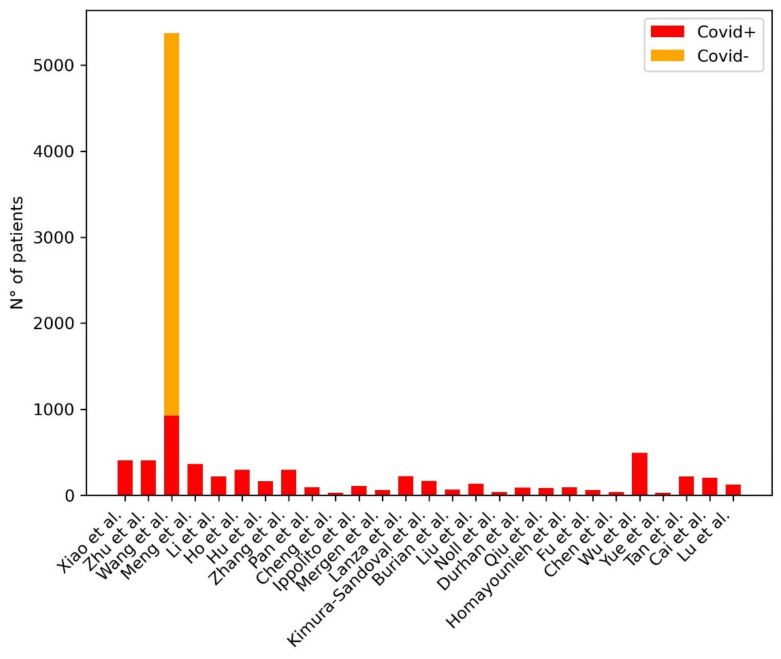
Distribution of subjects included in the studies for the development of ML models for the stratification and definition of severity and complications of COVID-19 pneumonia at Chest CT. The plot shows the distribution of the subjects included in the studies: in the legend in the right upper corner of the figure, the red bar represents the COVID-19 pneumonia group of patients, the yellow bar represents the non-COVID-19 pneumonia group of patients.

In Table 7, we provided a summary of the papers included in our review focused on AI in the stratification and definition of severity and complications of COVID-19 pneumonia at Chest CT. Figure 7 shows the distribution of subjects included considering those studies where it was clearly stated.

### 5.4. AI in the Differential Diagnosis of COVID-19 Pneumonia from Other Pneumonia at Chest CT 

The differentiation between pneumonia related to COVID-19 or to other pathogens represents a challenge due to superimposable clinical and radiological characteristics, but it is critical for early diagnosis and pandemic control.

Multiple studies have evaluated the diagnostic performance of different AI systems in the detection of COVID-19 and in the differential diagnosis with other common pneumonia, demonstrating an AUC in the range of 0.903 to 0.99 [128,129,130,131,132,133,134].

A Chinese retrospective and multi-center study developed a 3D DL model COVNet to detect COVID-19 and distinguish it from community-acquired pneumonia (CAP) due to typical and atypical bacteria or viruses. The calculated AUC for COVID-19 and CAP were 0.95 and 0.94, respectively, tested in a dataset of 3322 patients. The application of Gradient-weighted Class Activation Mapping (Grad-CAM) simplified the interpretability of the proposed model: it was an automatically generated heatmap that applied the red color to the suspected regions associated with the predicted class [133]. Other studies aimed to evaluate not only the performance of a proposed AI model in the differential diagnosis, but also the radiologist’s performance with and without AI assistance [131].

A retrospective study employed an EfficientNet architecture for the pneumonia classification task and a heatmap generated through a Grad-CAM for the visualization of the important image regions. The proposed model achieved an AUC of 0.95 and a higher accuracy, sensitivity and specificity than those of experienced radiologists (96% vs. 85%, 95% vs. 79%, 96% vs. 88%). Authors deduced that the performance of radiologists with AI assistance improved compared to manual interpretation, yielding higher accuracy (90%), sensitivity (88%), and specificity (91%) [133].

Another observation study by Zeng et al. tested a ML algorithm based on a radiomic texture analysis of CT imaging to distinguish pneumonia due to COVID-19 (NCP) and Influenza A (IAP). Their nomogram included 8 radiomic features as independent diagnosticators of NCP after application of LASSO regression model that were subsequently included into a radiomics score (higher values suggested COVID-related pneumonia). Their data suggested an excellent performance of the nomogram with an AUC of 0.87, helping clinicians in the choice of the right management [135].

**Figure 8:**
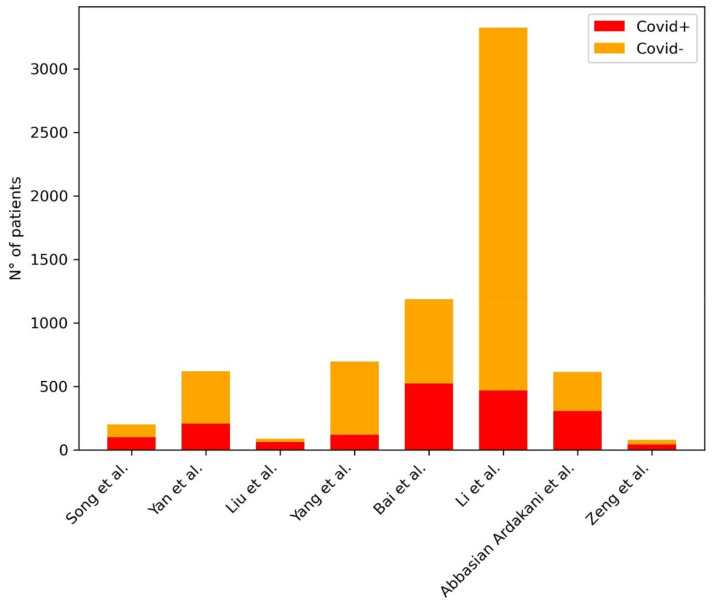
Distribution of subjects included in the studies for the development of ML models for the differential diagnosis of COVID-19 pneumonia from other pneumonia at Chest CT. The plot shows the distribution of the subjects included in the studies: in the legend in the right upper corner of the figure, the red bar represents the COVID-19 pneumonia group of patients, the yellow bar represents the non-COVID-19 pneumonia group of patients.

Table 8 provides a summary of the papers included in the review focused on AI in the differential diagnosis of COVID-19 pneumonia from other pneumonia at Chest CT. Figure 8 shows the distribution of subjects included considering those studies where it was clearly stated.

## 6. Computational Cost

A brief introduction to the concept of the computational cost is due. Computational cost is a generic name that refers to the computational power in (usually in terms of number of operations and memory) required to run an algorithm. Even the most demanding algorithms can be executed in reasonable time when more computational resources are provided. Generally speaking pipelines not based on deep learning have a rather low computational cost, both during training and inference. Indeed, studies based on radiomics and quantitative CT do not require expensive or very performant hardware to reach very low run times. Deep learning models, on the other hand, require modern, dedicated hardware (GPUs) to train in reasonable time but may still require multiple days to train.

This does not hinder their effectiveness or their use in production as the inference time is usually significantly lower. Among deep learning architectures some are designed specifically for a lower computational cost [136] while others focus on performance disregarding computational efficiency [137]. In particular, studies employing 3D convolutions [74] or studies that leverage multiple large models [81] are very computationally intensive and probably would require an amount of resources that few hospitals can provide. Nonetheless, for pipelines dedicated to a single disease, the required throughput is not too high and larger models can still provide value.

## 7. Discussion

In this literature review, we presented a structured review on the applications that AI can have in the clinical setting with regards to chest imaging in COVID-19 patients, describing the performances that the several DL/radiomics models have both in the identification, screening, stratification of patients as well as the differential diagnosis with other pneumonia.

Some of the previously described models showed very high performances, suggesting that the implementation of AI techniques would aid radiologists in their clinical practice, leading to a significant increase in accuracy values and leveraging their daily workflow performance.

However, the potential utility of the machine learning-based models using CXR and CT images for diagnostic and prognostic purposes in COVID-19 has been analyzed in a systematic review that included some of the previously discussed studies [21,84,91,98,99,121,132].

According to Roberts et al. [138], none of the included studies in their systematic review showed a sufficient robustness and reproducibility to be integrated into the regular clinical practice, due to biases in datasets, either too small or too heterogeneous, poor data integration or insufficient validation. In addition, some machine learning models may show over and under-fitting bias.

Specifically, as concerns the quality of the training data of the analyzed studies [138] the authors suggested the following key issues:

- a warning about using online repositories because of (1) the potential bias attributable to source issues and the inability to match demographics through populations (2) the possible overfitting on the shared dataset (3) the eventual low-resolution unbalanced across classes of the images of the shared dataset.
- to pay attention to CXRs projections (anteroposterior vs. posteroanterior) since models can wrongly correlate more severe disease to the view of the radiogram and not to the actual radiographic findings
- most studies did not report the timing between imaging and RT–PCR tests, since a negative RT–PCR test is a definitive exclusion criteria COVID-19 infection.

The authors recommended also major attention in the development of further ML-based algorithms; suggesting external validation, assessment with established frameworks (e.g., QUADAS, CLAIM, RQS) and checklists to identify these weaknesses [138].

Furthermore, other authors advised the sampling of large datasets to reduce predictive uncertainty, even though most works used small image samples, due to the lack of large open COVID-19 datasets (particularly for CXR) [139,140,141,142].

This is why further studies are needed to implement AI capacities in the above discussed settings (identification, screening, patients’ stratification and differential diagnosis), in order to guide the development of AI-empowered tools to reduce human error and assist radiologists in their decision-making process.

Limitations of the study:

Firstly, we would like to cite some limitations of the reviewed studies which include inadequate verification of datasets [138], limited time available considering the on-going pandemic, lack of large datasets for some authors. It’s worth mentioning that the first published work that reviews the usability of X-ray images to detect COVID-19 was of a very limited dataset [143]. In some investigations, the number of positive images used in the training was less than 100, which greatly limits the generalization power of the models, under the CNN paradigm [144]. The rapidly evolving and emerging applications of AL/ ML in COVID-19 can also represent another hurdle for reviewing the previous work. Some authors have managed to release newer versions of their early pandemic studies; enforcing their algorisms with larger datasets, including clinical information, overcoming some of the technical issues that was raised earlier such as over-fitting. Additionally, to avoid the limitations regarding the selection bias, we set a structured criteria for inclusion and exclusion of the selected studies.

## 8. Conclusions

The combination of chest imaging and artificial intelligence can help for a fast, accurate and precise disease extent quantification as well as for the identification of patients with severe short-term outcomes. AI/ ML as well as radiomics have feasible applications and optimistic potential to help leverage the radiologists’ workflow in the current pandemic. In other words, there are multiple domains that can benefit from AI applications in chest imaging, including identification, screening and risk stratification of COVID-19 cases. As aforementioned, the basic stages to tackle that pandemic include early and accurate identification of COVID-19, and ML can play a crucial role in this setting.

The integration of ML techniques will help in diagnosing this condition faster, cheaper, and safer in the upcoming years. However, various biases should be overcome in the development of further ML-based algorithms to guarantee sufficient robustness and reproducibility for their integration into clinical practice.

Though, as previously stated by Roberts et al. [138], many of those ML models developed could not be proved to be ready for the translation in clinical practice.

Datasets of higher quality, articles with enough documentation to be repeatable as well as external validation are required to give the currently developed ML models a sufficient robustness and reproducibility to integrate them into clinical practice.

## Figures and Tables

**Table 1:** AI in the identification of COVID-19 pneumonia at Chest X-ray.

Authors	Year	Population (No. of Patients)	ML Model	Results
Apostolopoulos et al.	2020	First dataset: 224 Covid+, 1204 covid-. plus a second dataset with 224 Covid+, 1218 Covid-	different CNNs (VGG19, MobileNet v2, Inception, Xception, Inception ResNet v2)	acc 96.78%, sen 98.66%, spe 96.46% (for binary class), acc 93.48% (for multi-class)
Ozturk et al.	2020	127 Covid+	DarkNet	acc 98.08%, sen 95.13%, spe 95.3%, (for binary class) acc 87.02%, sen 85.35%, spe 92.18%, (for multi-class)
Wang et al.	2020	358 Covid+, 13,604 Covid-	covid-net	acc 95%, sen 93%, spe 96% (for multi-class)
Borkowski et al.	2020	training: 484 Covid+, 1000 Covid-; validation: 10 Covid+, 20 Covid-	Microsoft custom vision	acc 97%, sen 100%, spe 95% (for binary)
Chowdhury et al.	2020	219 Covid+, 2659 Covid-	PDCOVID-net	acc 96.58%, pre 96.58%, rec 96.59%, F1 96.58% (for multi-class: covid, normal, viral pneumonia)
Toraman et al.	2020	231 Covid+ (1050 with data augmentation), 2100 Covid-	CapsNet	acc 89.48%, sen 84.22%, spe 92.11% (for multi-class: covid, normal, pneumonia)
Ouchicha et al.	2020	219 Covid+, 2686 Covid-	CVDNet	acc 97.79%, sen 96.83%, spe 98.02% (for multi-class: covid, normal, pneumonia)
Togacar et al.	2020	295 Covid+, 163 Covid-	MobileNet+squeezenet+SVM	acc 98.83%, sen 97.04%, spe 99.15% (for multi-class: covid, normal, pneumonia)
Hassantabar et al.	2020	315 Covid+, 367 Covid-	CNN and DNN	CNN: accuracy 93.2, sensitivity 96.1, DNN: accuracy 83.4, sensitivity 86
Mukherjee et al.	2021	Various datasets	CNN	Accuracy: 96.13

**Table 2:** AI in the screening of COVID-19 pneumonia at Chest X-ray.

Authors	Year	Population (No. of Patients)	ML Model	Results
Murphy et al.	2020	217 covid+, 237 covid-	CAD4COVID-XRay	AUC 0.81, specificity 85%
Wang et al.	2020	53 COVID+, 13,592 COVID-	covid-net	accuracy 92.4%
Narin et al.	2020	50 covid+, 50 covid-	ResNet-50, Inception V3, Inception-ResNet V2, ResNet101, ResNet152	accuracy 98% (ResNet-50)
Zhang et al.	2020	various datasets for internal and external validation	ResNet-18	sen 72.00%, spe 97.97%, AUC 95.18% (for binary class)
Xia et al.	2021	512 covid+, 106 covid-	DNN	AUC 0.919 (when combining cxr and clinical features: AUC 0.952, sensitivity 91.5, specificity 81.2)
Bassi et al.	2021	439 covid+, 1625 covid-	DenseNet201 and DenseNet121	accuracy 100

**Table 3:** AI in the stratification and definition of severity and complications of COVID-19 pneumonia at CXR.

Authors	Year	Population (No. of Patients)	ML Model	Results
Li et al.	2020	various datasets	convolutional siamese NN	AUC 0.80
Mushtaq et al.	2021	697 covid+	qXR	Achieving a statistical significance in predicting negative outcome in ED patients.
Zhu et al.	2020	131 covid+	VGG16	AI-predicted scores were highly correlated with radiologist scores

**Table 4:** AI in the differential diagnosis of COVID-19 pneumonia from other pneumonia at Chest X-ray.

Authors	Year	Population (No. of Patients)	ML Model	Results
Varela-Santos et al.	2021	various datasets (Cohen, Kermany)	FFNN, CNN	Various AUC values depending on the dataset/population/network considered
Jin et al.	2021	various datasets (NIH chext x ray database and others): 543 covid+, 600 covid-, 600 normal	hybrid ensemble model (AlexNet with ReliefF algorithm and SVM classifier)	accuracy 98.642, specificity 98.644, sensitivity 98.643, AUC 0.9997
Sharma et al.	2020	various datasets	CovidPred	accuracy 93.8
Tsiknakis et al.	2020	various datasets (Cohen, QUIBIM imagingcovid19): 137 covid+, 150 covid-, 150 normal	Inception-V3	sensibility 99, specificity 100, accuracy 100, AUC 1 for binary class (covid vs. other pneumonia)

**Table 5:** AI in the identification of COVID-19 pneumonia and its complications at Chest CT.

Authors	Year	ML Model	Population (No. of Patients)	Results
Anastasopoulos et al.	2020	U-Net	197 COVID+, 141 COVID-	Dice coefficient: 0.97
Yang et al.	2020	DenseNet	146 COVID+, 149 COVID-	AUC: 0.98
Harmon et al.	2020	AH-Net(segmentation) Densenet3D/2D+1 (classification)	922 COVID+, 1695 COVID-	AUC: 0.949—original design, 0.941—independent population
Ni et al.	2020	MVP-Net, 3D U-Net	14,435 (training): 2154 COVID+, 12,281 COVID- + 96 COVID+ (testing)	Accuracy: 82—per-lobe lung level, 0.94—per-patient level
Chen et al.	2020	U-Net++ with a ResNet50 backbone	106 (training and retrospective testing): 51 COVID+, 55 COVID- +27 (internal prospective testing): 16 COVID+, 11 COVID- +100 (external prospective testing): 50 COVID+, 50 COVID- 27 (internal prospec-tive testing): 16 COVID+, 11 COVID- + 100 (external pro-spective testing): 50 COVID+, 50 COVID-	Accuracy: 95.24—retrospective testing, 92.59—internal prospective testing, 96—external prospective testing
Zhang et al.	2020	QCT	2460 COVID+	Identification of lesions
Ma et al.	2020	QCT	18 COVID+	Identification of lesions and dynamic changes
Du et al.	2020	QCT	125 COVID+	Identification of lesions and dynamic changes
Lessmann et al.	2020	Two-stage U-Net (lobe segmentation and labeling), 3D U-net with nnU-Net framework (CT severity score prediction), 3D-inflated Inception (CO-RADS score prediction)	476 (training) 105 (internal test): 58 COVID+, 47 COVID- 262 (external test): 179 COVID+, 83 COVID-	AUC: 0.95—internal testing, 0.88—external testing
Liu et al.	2021	Radiomics	115 COVID+, 435 COVID-	AUC: 0.93
Fang et al.	2020	Radiomics	239 (training): 136 COVID+, 103 COVID- 90 (validation): 56 COVID+, 34 COVID-	AUC: 0.955
Chen et al.	2020	Radiomics	84 COVID+	AUC: 0.94
Voulodimos et al.	2020	FCN, U-net	10 COVID+	Unclear data: FCN Accuracy: ~0.9 (validation); Accuracy U-net: >0.9 (validation)
Sahood et al.	2021	U-net, SegNet	100—one slice CT scans	Accuracy: SegNet: 0.954; U-Net: 0.949
Mukherjee et al.	2021	CNN	336 COVID+, 336 COVID—(CXR + CT)	AUC CXR+CT: 0.9808 (AUC CT: 0.9731)

**Table 6:** AI in the screening of COVID-19 pneumonia at Chest CT.

Authors	Year	ML Model	Population (No. of Patients)	Results
Javor et al.	2020	ResNet50	209 COVID+, 209 COVID-	AUC: 0.956
Mei et al.	2020	LeNet, YOLO, DenseNet (pipeline developed in previous work)	419 COVID+, 486 COVID-	AUC: 0.92
Hermans et al.	2020	Logistic regression (no DL)	133 COVID+, 16 COVID-	AUC: 0.953

**Table 7:** AI in the stratification and definition of severity and complications of COVID-19 pneumonia at Chest CT.

Authors	Year	ML Model	Population (No. of Patients)	Results
Chatzitofis	2021	DenseNet201	497 COVID+	AUC: 0.79–0.97—moderate risk, 0.81–0.92—severe risk, 0.93–1.00—extreme risk
Xiao et al.	2020	Instance Aware ResNet34	408 COVID+	AUC: 0.892
Zhu et al.	2020	DL	408 COVID+	Accuracy: 85.91
Wang et al.	2020	DenseNet121-FPN (lung segmentation), COVID-19Net (novel) (COVID-19 diagnostic and prognostic analysis)	924 COVID+, 4448 COVID-	AUC-3 sets: 0.87, 0.88, 0.86
Meng et al.	2020	De-COVID19-Net (novel)	366 COVID+	AUC: 0.943
Li et al.	2020	DenseNet	46 COVID+	AUC: 0.93
Ho et al.	2021	Custom architectures (not very interesting) + an assortment of existing architectures	297 COVID+	AUC: 0.916
Hu et al.	2020	Custom architectures (not very interesting) + an assortment of existing architectures	164 COVID+	Identification of lesions
Li et al.	2020	QCT	196 COVID+	AUC: 0.97
Zhang et al.	2020	QCT	73 COVID+	Identification of volumes and dynamic changes
Pan et al.	2021	QCT	95 COVID+	Correlation with CT score—Spearman’s correlation coefficient 0.920
Cheng et al.	2020	QCT	30 COVID+	Significant correlation with laboratory data, PSI and CT score
Ippolito et al.	2020	QCT	108 COVID+	Significant correlation with laboratory data and CT score
Mergen et al.	2020	QCT	60 COVID+	Significant correlation with laboratory and clinical data
Lanza et al.	2020	QCT	222 COVID+	AUC: 0.83—oxygenation support, 0.86—intubation
Kimura-Sandoval et al.	2020	QCT	166 COVID+	AUC: 0.884—MV, 0.876—Mortality
Burian et al.	2020	QCT	65 COVID+	AUC: 0.79
Liu et al.	2020	QCT	134 COVID+	AUC: 0.93
Noll et al.	2020	QCT	37 COVID+	Correlation with clinical data
Durhan et al.	2020	QCT	90 COVID+	AUC: 0.902—severe pneumonia, 0.944—ICU admission
Wang et al.	2020	QCT	27 COVID+	Correlation with clinical data
Qiu et al.	2021	Radiomics	84 COVID+	AUC: 0.87
Homayounieh et al.	2020	Radiomics	92 COVID+	AUC: 0.99—disease severity, 0.90—outcome
Fu et al.	2020	Radiomics	64 COVID+	AUC: 0.833
Chen et al.	2021	Radiomics	40 COVID+	“AUC -3 classifiers: 0.82, 0.88,0.86, c-index-nomogram: 0.85”
Wu et al.	2020	Radiomics	492 COVID+	“AUC: 0.862—early-phase group, 0.976—late-phase group”
Li et al.	2020	DL-Radiomics	217 COVID+	AUC: 0.861
Yue et al.	2020	Radiomics	31 COVID+	AUC-2 models: 0.97, 0.92
Tan et al.	2020	Radiomics	219 COVID+	AUC-3 cohorts: 0.95, 0.95, 0.98
Cai et al.	2020	Radiomics	203 COVID+	AUC: 0.812
Lu et al.	2021	QCT	126 COVID+	AUC: 0.796—PLV, 0.783—PGV, 0.816—PCV
Zhang et al.	2020	QCT	294 COVID+	(Dice coefficients >0.85 and all accuracies >0.95)

**Table 8:** AI in the differential diagnosis of COVID-19 pneumonia from other pneumonia at Chest CT.

Authors	Year	ML Model	Population (No. of Patients)	Results
Song et al.	2020	BigBiGAN	98 COVID+, 103 COVID-	AUC: 0.972—internal test, 0.850—external validation
Yan et al.	2020	EfficientNetB0	206 COVID+, 412 COVID-	AUC: 0.962—per-slice, 0.934—per-scan
Liu et al.	2020	Radiomics	61 COVID+, 27 COVID-	AUC: 0.99
Yang et al.	2020	ResUNet	118 COVID+, 576 COVID-	AUC: 0.903
Bai et al.	2020	EfficientNet-B4	521 COVID+, 665 COVID-	AUC: 0.95—internal testing, 0.90—independent testing
Li et al.	2020	COVNet (novel)	468 COVID+, 2854 COVID-	AUC: 0.96
Abbasian Ardakani et al.	2021	COVIDiag	306 COVID+, 306 COVID-	AUC: 0.965
Zeng et al.	2020	Radiomics	41 COVID+, 37 COVID-	AUC: 0.87

## Data Availability

Not applicable.
